# Comprehensive reference intervals for white blood cell counts during pregnancy

**DOI:** 10.1186/s12884-023-06227-8

**Published:** 2024-01-05

**Authors:** Jinxiu Zhu, Zexin Li, Yuguo Deng, Liting Lan, Jinying Yang

**Affiliations:** 1 Department of Obstetrics, Longgang District Maternity and Child Healthcare Hospital of Shenzhen City, Shenzhen, Guangdong 518172 China; 2 Longgang Maternity and Child Institute of Shantou University Medical College, Shenzhen, Guangdong 518172 China; 3 Clinical Research Center, First Affiliated Hospital of Shantou University Medical College, Shantou, Guangdong 515041 China

**Keywords:** White blood cell, Leukocyte, Reference interval, Pregnancy, Pregnancy-related complications

## Abstract

**Background:**

White blood cell (WBC) count increases during pregnancy, necessitating reliable reference intervals for assessing infections and pregnancy-related complications. This study aimed to establish comprehensive reference intervals for WBC counts during pregnancy.

**Methods:**

The analysis included 17,737 pregnant women, with weekly WBC count measurements from pre-pregnancy to postpartum. A threshold linear regression model determined reference intervals, while Harris and Boyd’s test partitioned the intervals.

**Results:**

WBC count exhibited a significant increase during pregnancy, characterized by a rapid rise before 7 weeks of gestation, followed by a plateau. Neutrophils primarily drove this increase, showing a similar pattern. The threshold regression model and Harris and Boyd’s test supported partitioned reference intervals for WBC counts: 4.0–10.0 × 10^9/L for < = 2 weeks, 4.7–11.9 × 10^9/L for 3–5 weeks, and 5.7–14.4 × 10^9/L for > = 6 weeks of gestation. These reference intervals identified pregnant women with high WBC counts, who had a higher incidence of pregnancy-related complications including placenta previa, oligohydramnios, secondary uterine inertia, and intrauterine growth restriction.

**Conclusion:**

This study establishes comprehensive reference intervals for WBC counts during pregnancy. Monitoring WBC counts is clinically relevant, as elevated levels are associated with an increased risk of infection and pregnancy-related complications.

**Supplementary Information:**

The online version contains supplementary material available at 10.1186/s12884-023-06227-8.

## Introduction

The white blood cell (WBC) count, also known as leukocyte count, undergoes significant changes during pregnancy and the initial postpartum period. These changes are part of the body’s adaptation to accommodate the demands of the developing fetus. Specifically, the WBC count tends to increase from the first to the third trimester and peaks during the initial postpartum period [1]. Understanding these normal variations in WBC counts can assist clinicians in distinguishing between normal leukocytosis (an increase in the number of white blood cells) and pathological elevation of the WBC count during pregnancy and the initial postpartum period. This differentiation is crucial as it can prevent misdiagnosis of physiological leukocytosis as a bacterial infection, which could lead to unnecessary medication use that may harm the fetus.

In addition, a study found that leukocytosis (> 13.8 × 10^9/L) during the first trimester of pregnancy is significantly associated with an increased risk for obstetrical complications. These complications include preterm delivery before 37 weeks, hypertensive disorders, gestational diabetes mellitus, and cesarean section. Furthermore, women with leukocytosis during the first trimester had significantly higher rates of fetuses who were small for gestational age and with birth weight less than 2,500 g [2].

The findings of these studies indicate that the currently used reference interval (RI) for WBC count during pregnancy, based on the non-pregnant range (4.0–10.0 × 10^9/L) in China, [3] is inadequate for distinguishing infection and alerting for pregnancy-related complications. Previous studies have reported varying upper limits of the RI during pregnancy, ranging from 13.8 to 19.6 × 10^9/L. However, these studies were conducted on smaller populations, with differences in ethnicity and gestational age at the time of sampling [4–8]. A large-population study of 24,318 pregnant women in Oxford, UK have mapped the trajectory of WBC between 8 and 40 weeks of gestation and defined 95% RI for total WBC as 5.7–15 × 10^9/L [9]. However, the RI within the 0–7 weeks of gestation was not investigated. The main objective of this study was to define pregnancy-specific RI for WBC count and assess their ability to detect pregnancy-related complications.

## Methods

### Study population and design

A retrospective longitudinal study was conducted at Shenzhen Longgang Maternity and Child Health Hospital, utilizing data from deliveries that occurred between June 2020 and March 2022. During this period, the equipment and reagents for blood routine test were consistent, and testing was recommended at under 13 weeks, 16–20 weeks, 24–28 weeks, 30–32 weeks, and around 37 weeks of pregnancy. The inclusion criteria were as follows: patients who were registered, examined, and delivered at our hospital and had at least one blood routine test, not in the week before laboring. Blood routine tests conducted within the week before laboring probably affected by some conditions that can initiate labor, such as premature rupture of membranes, or by the administration of certain medications like steroids. Exclusion criteria included: age < 18 years old; lasting infectious diseases such as human immunodeficiency virus infection, syphilis, hepatitis B or C; immune diseases such as systemic lupus erythematosus, Sjögren’s syndrome, ankylosing spondylitis, and antiphospholipid syndrome; a history of malignant or borderline tumors; other diseases affecting blood cells such as thalassemia and glucose-6-phosphate dehydrogenase; more than ten blood tests during pregnancy that increased likelihood that these were taken to investigate an abnormality; incomplete blood routine data; extreme outliers that lay more than three times the interquartile range below the first quartile or above the third quartile [10].

### Data collection

Demographic, clinical, and laboratory data were collected from electronic hospital records in this study. Demographic data encompassed information such as ethnicity, age, height, and weight. Height and weight measurements were taken during registration, primarily between 6 and 12 gestational weeks, and were used to calculate body mass index (BMI). Clinical data included variables such as blood pressure, gestational age at delivery, baby sex, gravidity, parity, delivery style, and labor-related diagnoses. Laboratory data consisted of blood routine tests performed as part of routine clinical care. Venous blood samples were collected using 4.5 mL potassium EDTA tubes and analyzed using the Sysmex XS-500i hematology analyzer (Sysmex Corporation Kobe, Japan). The same analytical method was consistently employed throughout the study period. Gestational age was recorded in weeks, including the corresponding weeks and days (e.g., 37 weeks represents a period from 37 to 37^+ 6^ weeks), and categorized into three stages: first trimester (0 to 13 weeks of gestation), second trimester (14 to 27 weeks), and third trimester (28 to 42 weeks). Additionally, samples during the prepregnancy stage (0 to 11 weeks prior to pregnancy) and the postpartum stage (0 to 11 weeks following delivery) were collected for the comparison with WBC trends during pregnancy.

### Statistical analysis

Statistical analysis was performed using R software in this study. Descriptive statistics were used to summarize the demographic and clinical characteristics of the study samples. Continuous variables were presented as means with standard deviations (SD) or medians with interquartile ranges (IQR), depending on their distribution. Categorical variables were reported as frequencies and percentages.

In the analysis of the trend of WBC count, median was used due to the non-normal distribution of the data (Figure S2), and Wilcoxon tests with Bonferroni correction was used to compare WBC levels between different gestational stages.

To achieve a normal distribution and maintain the trend of WBC count across pregnancy, WBC count was log-transformed (Figure S3). A threshold regression model with a segmented-type change point was used to fit the log-transformed WBC count against gestational age. Due to the uneven sample size across different gestational ages, bootstrapping with 100 replications was employed for resampling. Each replication included 120 samples per gestational week. The median values of the model parameters were extracted from the bootstrap results to construct the regression equation, and the mean log-transformed WBC count for each gestational week was calculated. The residuals of the model across different gestational ages showed minimal deviation (Figure S4), allowing us to utilize the residual standard deviation (RSD) as the standard deviation of WBC count for each gestational week. The gestational age-specific RIs for log-transformed WBC count were calculated as the mean ± 1.96 standard deviations. Finally, the RIs for log-transformed WBC count were transformed back to RIs for WBC count.

Harris and Boyd’s test, which is recommended by the National Committee for Clinical Laboratory Standards (NCCLS), was used to determine the partitioning of RI during pregnancy [11]. Harris and Boyd’s test is composed of two independent partitioning tests. The first one uses the standard normal deviate score *z* as a test parameter: *z* = [(‾x_2_ -‾x_1_)/(s_1_^2^/n_1_ + s_2_^2^/n_2_)]^1/2^, where ‾x _i_, s_i_, and n_i_ are the mean, standard deviation, and sample size, respectively, of subgroup i. Mean and standard deviation were from the threshold regression model. When *z* score > = 5[(n_1_ + n_2_)/240]^1/2^, a reference interval partitioning was required. The second one is if the ratio between standard deviations > = 1.5, separate reference intervals are recommended for the subgroups, even if the means are equal. In this study, the second test was not applicable due to equal standard deviations.

In the analysis of the association between high WBC and pregnancy-related complications, we conducted an analysis of the diagnoses for all subjects and selected the most prevalent pregnancy-related complications for further investigation. Utilizing the RIs proposed in our study, we employed them to identify pregnant women with high WBC counts. Women with at least one test result above the gestational age-specific RI were categorized into the High group, while those with all test results within the RI were classified into the non-High group. The association between WBC count and pregnancy-related complications was assessed using chi-square tests or Fisher’s exact test, depending on the expected frequencies.

All statistical tests were two-tailed, and a significance level of *P* < 0.05 was considered statistically significant.

## Results

### Overview of the study population

**Fig. 1:**
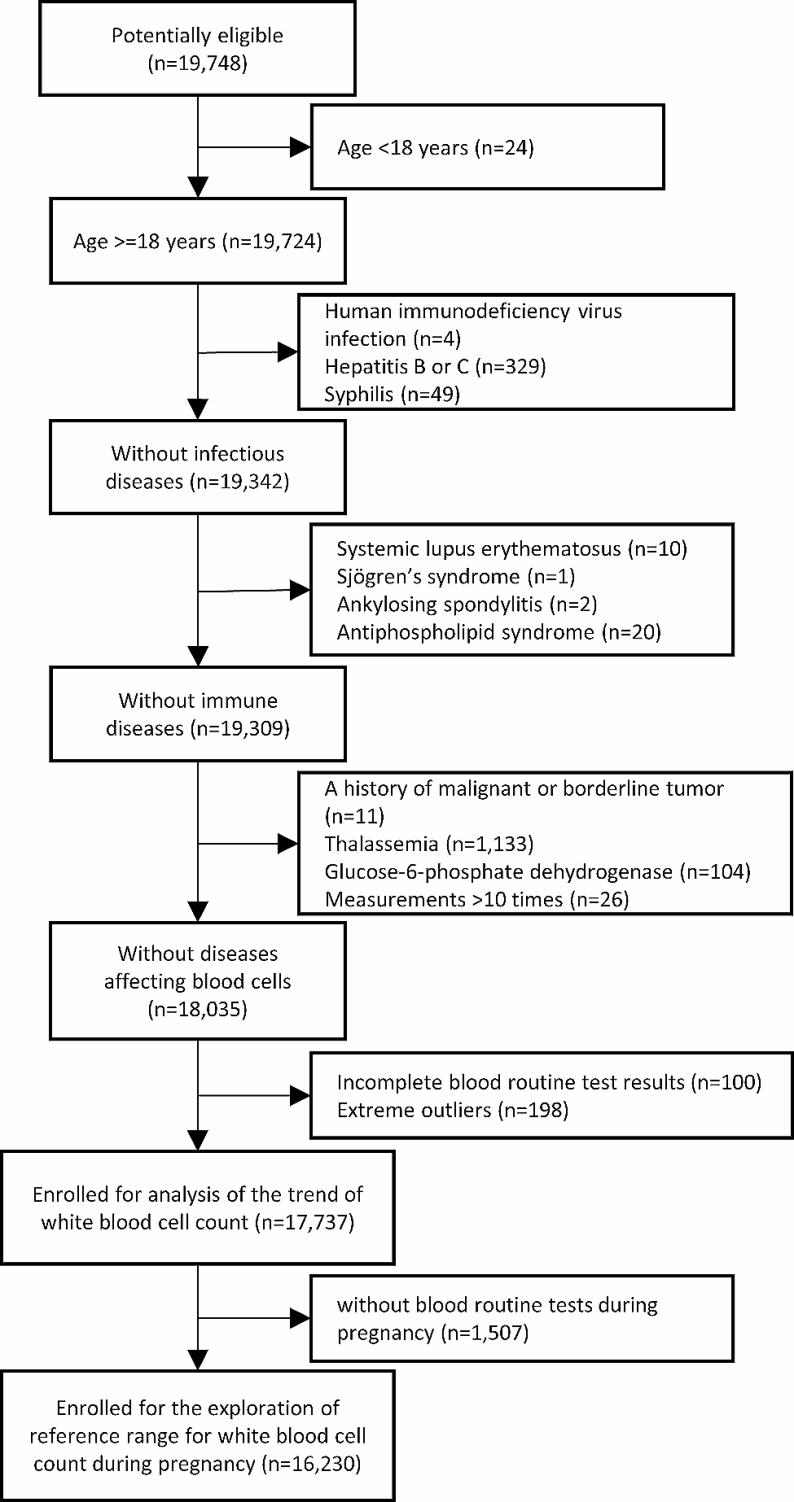
Flowchart of the study design

In this study, a total of 19,748 deliveries were initially identified to meet the inclusion criteria. Subsequently, women with age less than 18 years old, infectious diseases, immune diseases, a history of malignant or borderline tumors, other diseases impacting blood cells, incomplete blood routine data, and extreme outliers were excluded from the analysis. As a result, 17,737 pregnancies were included for the analysis of the WBC trend, as illustrated in Fig. 1. The majority of the study population consisted of ethnic Han individuals (97.1%), and the average age was 30.2 ± 4.48 years. Further characteristics of the population are detailed in Table S1.

### White blood cell increased during pregnancy

The WBC count was assessed in this study at a median of five times, with an interquartile range of 3–6, resulting in a total of 86,578 results. Most of the samples were collected according to the recommended schedule, with notable peaks observed at 8 weeks (n = 2,124), 16 weeks (n = 6,103), 24 weeks (n = 5,035), 30 weeks (n = 5,188), and 36 weeks (n = 7,755) of gestation, as well as at 0 week (n = 9,526) and 4 weeks (n = 4,811) of postpartum (Figure S1).

**Fig. 2:**
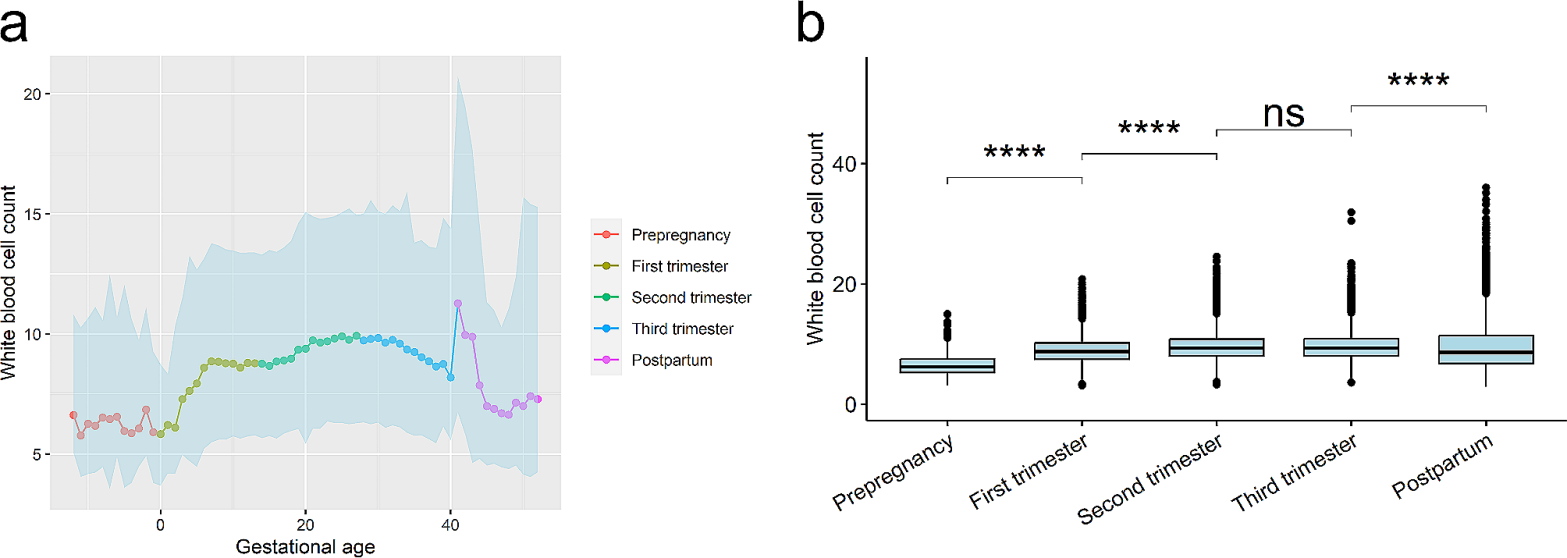
Trend of white blood cell count across prepregnancy, pregnancy, and postpartum. (**a**) Displays the median and 95% confidence interval (light blue zone) for white blood cell count in each week. Gestational age was recorded as weeks, representing the corresponding weeks and days (e.g., 37 weeks means 37 to 37^+ 6^ weeks). (**b**) Compared the median white blood cell count between each two adjacent stages. Wilcoxon test was used for the comparison. ns means *P* > = 0.05 and **** means *P* < 0.0001

It is widely recognized that WBC levels increase during pregnancy. This study confirms this trend by analyzing data from the prepregnancy period to the postpartum phase. The median WBC count remained relatively stable at around 6 × 10^9/L before pregnancy and increased after conception (Fig. 2a). A notable surge in WBC count was observed during the first 7 weeks of gestation, followed by a relatively steady trend until the 15 weeks. Subsequently, it gradually increased, reaching a peak of 9.9 × 10^9/L at 25 weeks, followed by a gradual decline until 40 weeks. The increase in WBC count during the first and second trimesters was statistically significant (all P values < 0.001), rising from 6.25 × 10^9/L in the pre-pregnancy phase to 8.73 × 10^9/L in the first trimester and 9.33 × 10^9/L in the second trimester (Fig. 2b). WBC count in the third trimester was similar to that in the second trimester, measuring 9.35 × 10^9/L. An intriguing observation was noted during the postpartum period, where the WBC count rapidly increased to its highest level of 11.3 × 10^9/L in the immediate postnatal week (0 to 6 days). Following this peak, the WBC count gradually declined, reaching a level of 7.0 × 10^9/L at 4 weeks postnatally, which was similar to the WBC count in the prepregnancy phase. Subsequently, the WBC count remained relatively consistent.

**Fig. 3:**
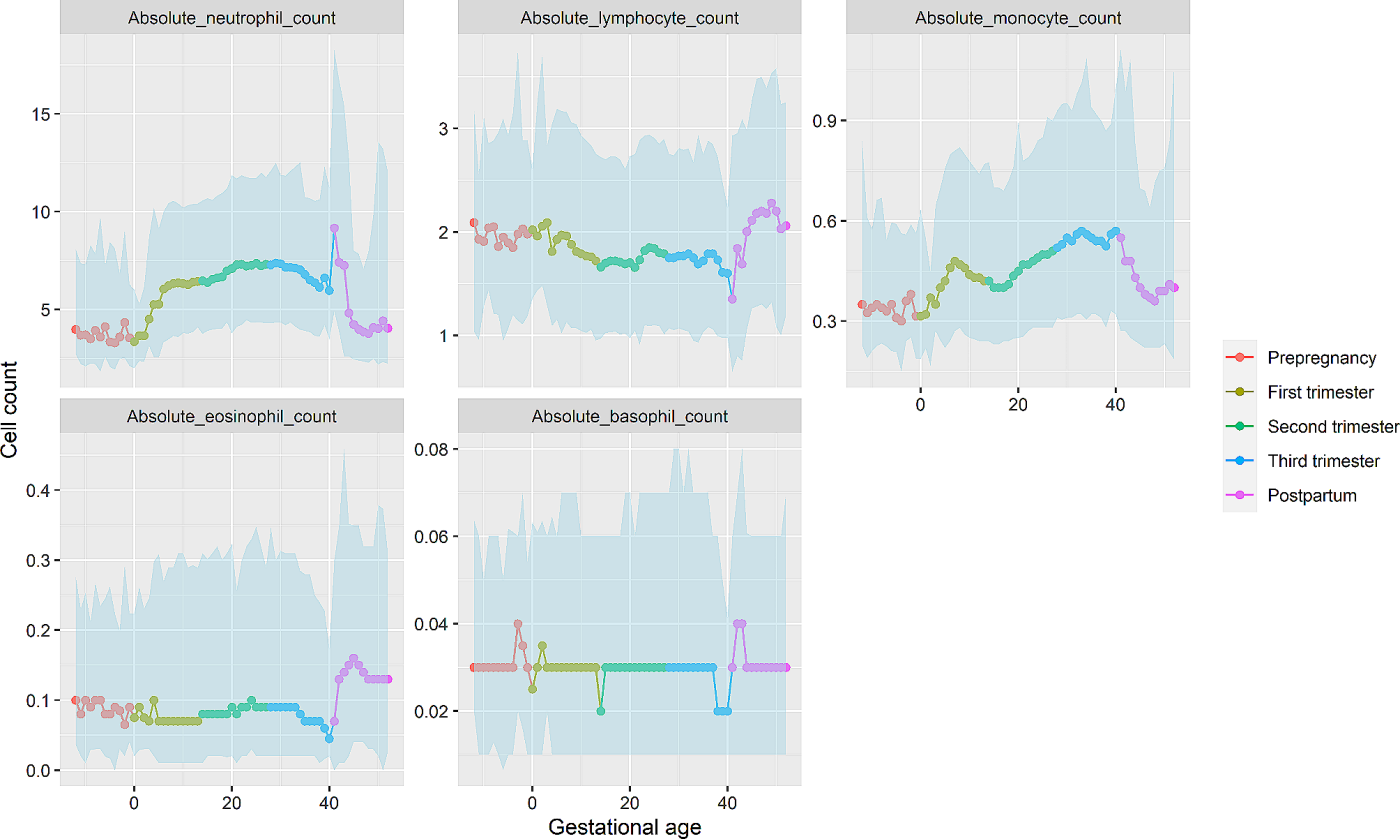
Trends in subtypes of white blood cell across prepregnancy, pregnancy, and postpartum. The median and 95% confidence interval (light blue zone) in each week were displayed

The elevation of white blood cell (WBC) count during pregnancy is primarily attributed to an increase in neutrophils, which exhibit a similar trend throughout gestation. Monocytes also experience an increase during pregnancy, albeit to a lesser extent, as their absolute count is relatively low. Interestingly, lymphocytes display an opposite pattern compared to neutrophils. They decline during the first trimester and remain relatively stable during the second and third trimesters. Following childbirth, lymphocytes experience a sharp decline within the initial week, reaching their lowest level, and subsequently begin to increase, gradually returning to prepregnancy levels during the 3–4 weeks postnatally. Eosinophils and basophils, on the other hand, do not exhibit significant variations during pregnancy (Fig. 3).

### Estimation of reference interval of white blood cell

**Fig. 4:**
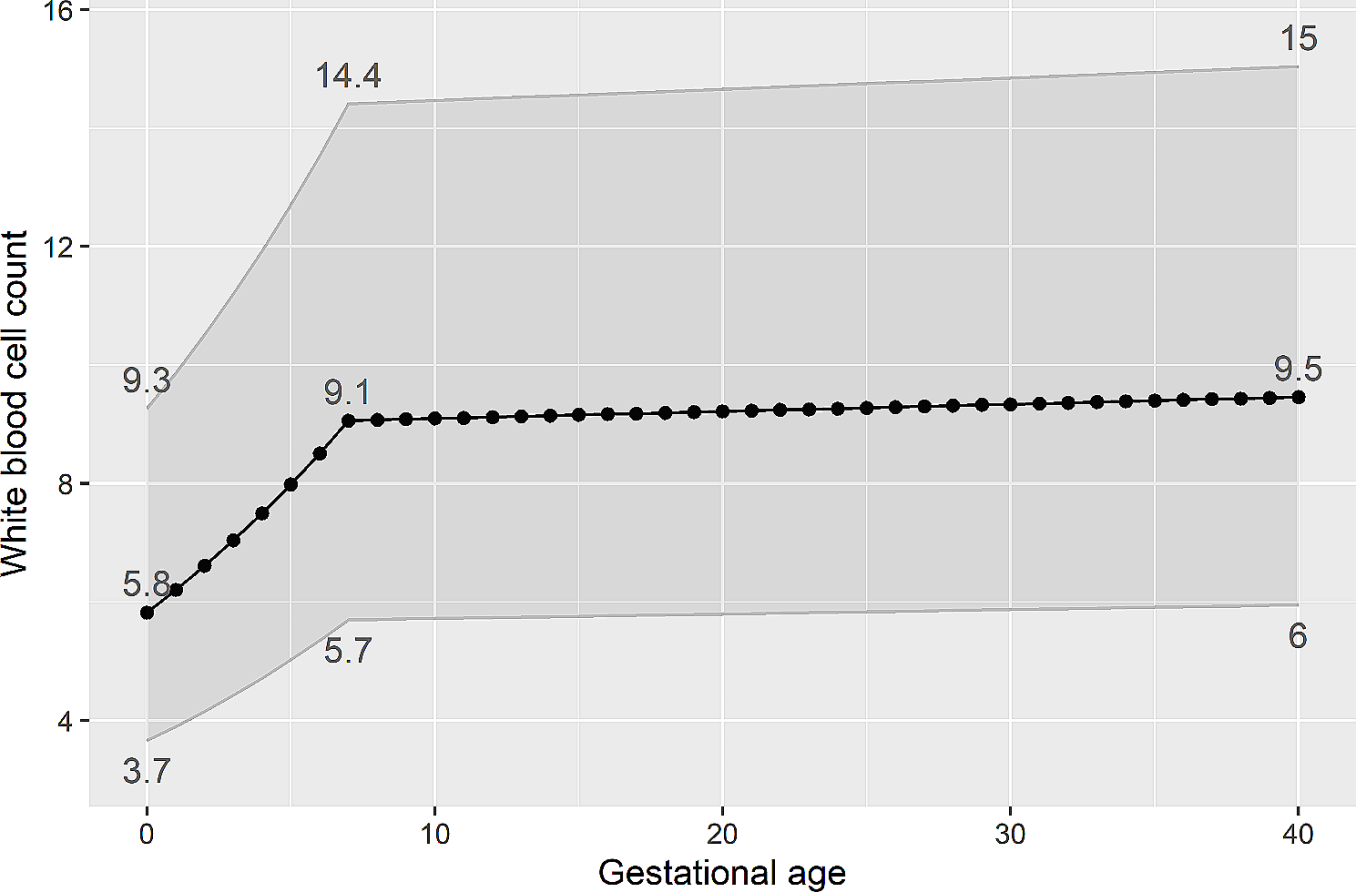
Means and reference intervals for each week of gestation. Means and reference intervals were calculated based on a threshold regression model. The grey zone shows reference intervals. Values of means and reference intervals for the 0th, 7th, 40th week were annotated

To determine the RI for WBC count, we included samples obtained from 0 to 40 weeks of gestation, comprising a total of 16,230 pregnant women in this analysis. Notably, a turning point was observed at 7 weeks of gestation. A regression model as well identified a threshold at 7 weeks, prior to which WBC count exhibited a rapid increase from 5.8 to 9.1 × 10^9/L, and subsequently, a slower increase from 9.1 to 9.5 × 10^9/L. The upper limit progressively increased from 9.3, 14.4, to 15 × 10^9/L at 0, 7, and 40 weeks, respectively. Likewise, the lower limit increased from 3.7, 5.7, to 6 × 10^9/L at 0, 7, and 40 weeks, respectively (Fig. 4). The upper and lower limits of all gestational weeks were presented in Table S2.

As Table 1 shows, we partitioned the reference intervals for 4 and 7 weeks of gestation. However, the difference in RIs between 7 and 40 weeks of gestation was insufficient for further partitioning, indicating that the reference interval of 5.7–14.4 × 10^9/L is suitable for the gestational period from 7 to 40 weeks. Within the first 7 weeks of gestation, the increase of one or two weeks did not warrant partitioning of the RIs according to the Harris and Boyd’s test. Therefore, we suggest that RIs for < = 2 weeks of gestation can utilize the non-pregnancy RI of 4.0–10.0 × 10^9/L. Reference intervals for 3–5 weeks of gestation can employ the range of 4.7–11.9 × 10^9/L, while > = 6 weeks of gestation can utilize the range of 5.7–14.4 × 10^9/L.

**Table 1:** Application of the Harris and Boyd partition criteria for pregnancy age

Pregnancy age	Reference interval (×10^9/L)	*z* score
0 week	3.7–9.3	
4 weeks	4.7–11.9	8.27*
7 weeks	5.7–14.4	6.20*
40 weeks	6.0–15.0	1.41

Z score was calculated by the formular [(‾x_2_ -‾x_1_)/(s_1_^2^/n_1_ + s_2_^2^/n_2_)]^1/2^, x_2_ and x_1_ are the adjacent points in 0, 4, 7, 40 weeks of gestation. When *z* score > = 5[(n_1_ + n_2_)/240]^1/2^, a reference interval partitioning was required. *Reference interval partitioning was necessary according to the *z* score from the Harris and Boyd’s test

### Sensitivity analysis

To allow for a more comprehensive evaluation of RI for WBC count during pregnancy, four different approaches for establishing RIs were compared in this study: non-pregnant 95% RI, pregnant 95% RI using threshold regression, parametric pregnant 95% RI, and non-parametric pregnant 95% RI (Table 2). The currently utilized RI for pregnancy is based on the non-pregnant range of 4–10 × 10^9/L, with both the upper and lower limits lower than those established by the other three methods. The new reference interval for > = 6 weeks of gestation based on the threshold regression (5.7–14.4 × 10^9/L) was similar to the parametric (5.9–14.5 × 10^9/L) and non-parametric (5.9–14.4 × 10^9/L) methods, indicating the robustness of the results. However, the RI for 3–5 weeks was smaller in the upper limit (4.7–11.9 vs. 4.6–13.2 vs. 4.6–12.9 × 10^9/L), probably due to the impact of low sample size.

**Table 2:** Reference intervals for white blood cell count during pregnancy

Methods	RI for 0–2 weeks†	RI for 3–5 weeks	RI for >=6 weeks
Non-pregnant 95% reference Intervals (×10^9/L)	4.0–10.0	4.0–10.0	4.0–10.0
Pregnant 95% reference intervals based on threshold regression (suggested) (×10^9/L)	4.0–10.0	4.7–11.9	5.7–14.4
Parametric pregnant 95% reference intervals (×10^9/L)	3.8–9.8	4.6–13.2	5.9–14.5
Non-parametric pregnant 95% reference intervals (×10^9/L)	3.9–9.1	4.6–12.9	5.9–14.4

†RI, reference interval

Additionally, the study compared the difference between threshold and linear regressions for establishing RIs. Given the limited sample size in the 0 to 6 weeks gestation period and a changing point at 7th week, this comparison was conducted using samples from 7 to 40 weeks gestation. As depicted in Figure S5, the upper limits in the threshold regression model were only slightly higher than those obtained through linear regression.

### High white blood cell count is associated with pregnancy-related complications

As demonstrated in Table 3, women in the high WBC group exhibited a significantly increased risk of placenta previa by 111% (P = 0.003), oligohydramnios by 46% (P = 0.029), secondary uterine inertia by 32% (P = 0.027), and intrauterine growth restriction by 73% (P = 0.032). Furthermore, within the complicated cases, the High group exhibited a higher proportion of women experiencing one, three, and four complications (Figure S6).

**Table 3:** Comparisons of complications between pregnant women with high and non-high white blood cell count

Variable	Non-high *n* = 15,244	High *n* = 986	OR [95% confidence interval]	*P* value
Age (years), mean (SD)†	30.2 (4.44)	29.3 (4.29)	\	< 0.001***
Placental abruption, n (%)	68 (0.45%)	4 (0.41%)	0.94 [0.28;2.29]	1.000
Placenta previa, n (%)	149 (0.98%)	20 (2.03%)	2.11 [1.28;3.30]	0.003**
Placental adhesion, n (%)	441 (2.89%)	38 (3.85%)	1.35 [0.95;1.87]	0.103
Premature rupture of membranes, n (%)	2766 (18.1%)	201 (20.4%)	1.16 [0.98;1.35]	0.085
Oligohydramnios, n (%)	440 (2.89%)	41 (4.16%)	1.46 [1.04;2.01]	0.029*
Polyhydramnios, n (%)	122 (0.80%)	9 (0.91%)	1.16 [0.54;2.16]	0.842
Gestational hypertension, n (%)	239 (1.57%)	21 (2.13%)	1.38 [0.85;2.11]	0.218
Preeclampsia or eclampsia, n (%)	240 (1.57%)	23 (2.33%)	1.50 [0.95;2.27]	0.090
Gestational diabetes, n (%)	2993 (19.6%)	189 (19.2%)	0.97 [0.82;1.14]	0.752
Intrahepatic cholestasis of pregnancy, n (%)	419 (2.75%)	23 (2.33%)	0.85 [0.54;1.27]	0.499
Secondary uterine inertia, n (%)	931 (6.11%)	78 (7.91%)	1.32 [1.03;1.67]	0.027*
Postpartum hemorrhage, n (%)	262 (1.72%)	18 (1.83%)	1.07 [0.64;1.69]	0.902
Hypothyroidism in pregnancy, n (%)	485 (3.18%)	36 (3.65%)	1.16 [0.81;1.61]	0.473
Hyperthyroidism in pregnancy, n (%)	78 (0.51%)	5 (0.51%)	1.02 [0.35;2.29]	1.000
Premature birth, n (%)	513 (3.37%)	42 (4.26%)	1.28 [0.92;1.75]	0.159
Low birth weight, n (%)	389 (2.55%)	35 (3.55%)	1.41 [0.98;1.98]	0.072
Macrosomia, n (%)	503 (3.30%)	25 (2.54%)	0.77 [0.50;1.13]	0.223
Intrauterine growth restriction, n (%)	182 (1.19%)	20 (2.03%)	1.73 [1.05;2.68]	0.032*
Fetal distress, n (%)	343 (2.25%)	29 (2.94%)	1.32 [0.88;1.91]	0.195
Fetal malformation, n (%)	42 (0.28%)	1 (0.10%)	0.42 [0.02;1.90]	0.519
Complications, n (%)‡	9538 (62.6%)	646 (65.5%)	1.14 [0.99;1.30]	0.068

†SD: standard deviation

‡Complications were women with one or more pregnancy-related complications

*P < 0.05

**P < 0.01

***P < 0.001

## Discussion

This study analyzed white blood cell (WBC) trends in a large population of 17,737 pregnant women, confirming increased WBC levels during pregnancy, primarily due to neutrophils. In a subpopulation of 16,230 pregnant women, results suggested RIs for < = 2 weeks, 3–5 weeks, and > = 6 weeks of gestation can utilize the range of 4–10 × 10^9/L, 4.7–11.9 × 10^9/L, and 5.7–14.4 × 10^9/L, respectively. Pregnant women with WBC over the upper limits had higher risk in certain pregnancy complications. These insights can help improve health monitoring and risk assessment during pregnancy.

WBC count, particularly neutrophil count, is known to increase during pregnancy, a phenomenon termed “physiologic leukocytosis of pregnancy”. Several factors contribute to this phenomenon. Hormones such as estrogen and cortisol, elevated during pregnancy, stimulate the bone marrow to produce more WBCs. Additionally, these hormones prolong the lifespan of neutrophils by inhibiting their apoptosis, leading to an increased number of circulating neutrophils. Pregnancy itself induces a stress state, triggering the release of stress hormones like cortisol and catecholamines, which can further stimulate WBC production and release from the bone marrow. The mild systemic inflammatory state associated with pregnancy also promotes the production of certain cytokines that drive WBC production [12, 13]. Following labor, the rise in WBC count is a normal and beneficial response to the stress of childbirth. This increase serves to protect the mother from infections and support the healing process. The release of inflammatory mediators during labor and the tissue trauma associated with childbirth contribute to the elevation in WBC count. Moreover, the presence of bacteria in the birth canal can also trigger this response. The rapid increase in WBC count is considered a protective mechanism [14, 15]. Notably, the phenomenon of WBC increase during pregnancy and its subsequent peak after delivery has been observed in previous studies as well, confirming its consistency and significance [1, 9].

Although several studies have explored the RI of WBC count during pregnancy, [1, 5, 7–9, 16–20] there are notable limitations that cannot be overlooked. Firstly, many of these studies were based on small populations, which may not provide robust and reliable results. Secondly, some studies only sampled specific gestational weeks or trimesters, failing to capture the entire gestational process. Thirdly, most studies did not consider the necessity of RI partitioning, instead focusing solely on providing the 95% confidence interval. Akkaya et al. conducted a study involving 40,325 pregnant women with 82,786 complete blood count evaluations from 6 to the 41 weeks of gestation. They reported the 3rd, 5th, 10th, 50th, 95th and 99th percentile values for total and differential leukocyte counts according to trimester. While this study encompassed a large-scale population and a wide range of gestational ages, the clinical applicability of the results may be limited due to the specific percentile values chosen that were not the 2.5th and 97.5th percentile [1]. Another large-scale study conducted by Dockree et al. included 24,318 pregnant women with 80,637 samples from 8 to 40 weeks of gestation, and RI was determined as 5.7–15.0 × 10^9/L. The authors confirmed the need for a pregnancy-specific RI, but refutes the need for partitioned, gestational-age specific limits [9]. The results were similar to ours, in which RI was suggested as 5.7–14.4 × 10^9/L when gestational age > = 6 weeks.

In previous studies, the estimation of WBC count before 6 weeks of gestation was often neglected due to the low likelihood of detecting pregnancy during this early period. In our study, which involved a large population, the sample size for gestational age < 5 weeks did not reach the minimum requirement of at least 120 participants needed for accurate estimation of reference limits [21]. Therefore, we employed bootstrapping to even the sample size as 120 at each gestational week and a threshold regression model to fit the means, using the residual standard deviation as the standard deviation for calculating the RIs. The approaches help reduce variation resulting from the small sample size. The threshold regression model proved to be as robust as linear regression (Figure S5), while also accommodating data with changing points. The impact of the low sample size is evident in Table 2, where the RIs for gestational age > = 6 weeks estimated by three different methods were similar. However, for the 2–5 weeks gestation period, the upper limits varied among the methods. Dockree et al., who only included women with gestational age over 8 weeks, concluded that RI partitioning was unnecessary [9]. However, when we included gestational age before 8 weeks, an intriguing finding emerged. A significant turning point was identified in the 7th week, with WBC count increasing from a median of 5.8 to 9.1 × 10^9/L before that point (Fig. 4). According to the Harris and Boyd’s test, RI partitioning was warranted in the 4th week, resulting in the range of 4.7–11.9 × 10^9/L. Furthermore, our analysis revealed that within the first 7 weeks of gestation, the progression of one or two weeks refuted the need for RI partitioning.

Based on these observations, we propose the following RIs: <=2 weeks of gestation can utilize the non-pregnancy reference interval of 4–10 × 10^9/L. For 3–5 weeks of gestation, the reference interval can be set as 4.7–11.9 × 10^9/L, while > = 6 weeks of gestation can utilize the range of 5.7–14.4 × 10^9/L. These recommendations provide more accurate and appropriate RIs for WBC count during different weeks of gestation.

During pregnancy, the information carried by blood cells is beyond that found in non-pregnant individuals. Elevated levels of haemoglobin concentration has been associated with adverse maternal and neonatal outcomes [22], and similarly, a high WBC count is also linked to the adverse outcomes. A retrospective study showed that total leukocyte count, neutrophil count, and neutrophil-to-lymphocyte ratio were significantly higher in the placenta previa group compared to the controls, suggesting a valuable predictor for placenta previa [23]. A study suggests that higher total WBC and absolute neutrophil counts in the third trimester are associated with small-for-gestational-age birth. These associations may indicate a cycle of inflammation and placental dysfunction contributing to fetal growth restriction [24]. In our study, we as well found the WBC count over upper limit was associated with the placenta previa and fetal growth restriction. Moreover, we discovered additional associations between high WBC count and complications such as oligohydramnios and secondary uterine inertia, which have not been previously reported. The potential mechanisms for these observations might be attributed to systemic chronic inflammation, which can lead to alterations in the uterine environment [25]. These novel findings suggest that establishing an RI for WBC during pregnancy is crucial not only for detecting infections but also for identifying and monitoring various pregnancy-related complications.

In conclusion, our study examined WBC trends in a large population of pregnant women and confirmed the well-known increase in WBC levels during pregnancy, primarily driven by neutrophils. Our findings suggest that different RIs should be applied based on gestational age, with RI partitioning necessary for specific periods. We also identified associations between high WBC count and various pregnancy-related complications, including placenta previa, fetal growth restriction, oligohydramnios, secondary uterine inertia, and shoulder presentation. These results highlight the importance of using appropriate RIs for WBC count during pregnancy to enhance health monitoring and risk assessment.

## Strength and limitation

The strengths of this study lie in its extensive coverage of a large population, encompassing the prepregnancy, pregnancy, and postpartum periods. This comprehensive monitoring of WBC count provides a panoramic view of WBC trends throughout the entire pregnancy journey, ensuring robust and generalizable findings. The utilization of a threshold regression model to establish gestational week-specific RIs for WBC count addresses the limitations of previous studies and improves the accuracy of interpretation. The implementation of partitioned RIs for WBC count demonstrates high clinical applicability and translational potential. Furthermore, the identification of associations between high WBC count and various pregnancy-related complications contributes valuable insights to the existing scientific knowledge. Nonetheless, limitations of the study include its single-center nature and the homogeneity of the racial composition, potentially introducing biases. Additionally, the limited sample size of less than 120 participants per week before 5 weeks of gestation may constrain the generalizability of the RI established for early pregnancy. Finally, our study has excluded many, but not all, diseases and medical conditions known to affect WBC count.

### Electronic supplementary material

Below is the link to the electronic supplementary material.

Supplementary Material 1

## Data Availability

Individual participant data may be requested from the corresponding author, after de-identification of data underlying the text, tables, and figures presented in this article.

## Abbreviations

WBC: white blood cell
RI: reference interval
BMI: body mass index
