# Supplementary material for: Comprehensive reference intervals for white blood cell counts during pregnancy
Source: BMC Pregnancy Childbirth. 2024 Jan 5;24:35. doi: 10.1186/s12884-023-06227-8 (PMC10768452; doi:10.1186/s12884-023-06227-8)
Supplement: Supplementary file 1 — Supplementary Material 1 [file 12884_2023_6227_MOESM1_ESM.docx]

Figure S1. Frequency distribution of sampling for blood routine test. Sample size of blood test from prepregnancy (12 weeks before pregnancy) to postpartum (12 weeks after labor) were displayed.


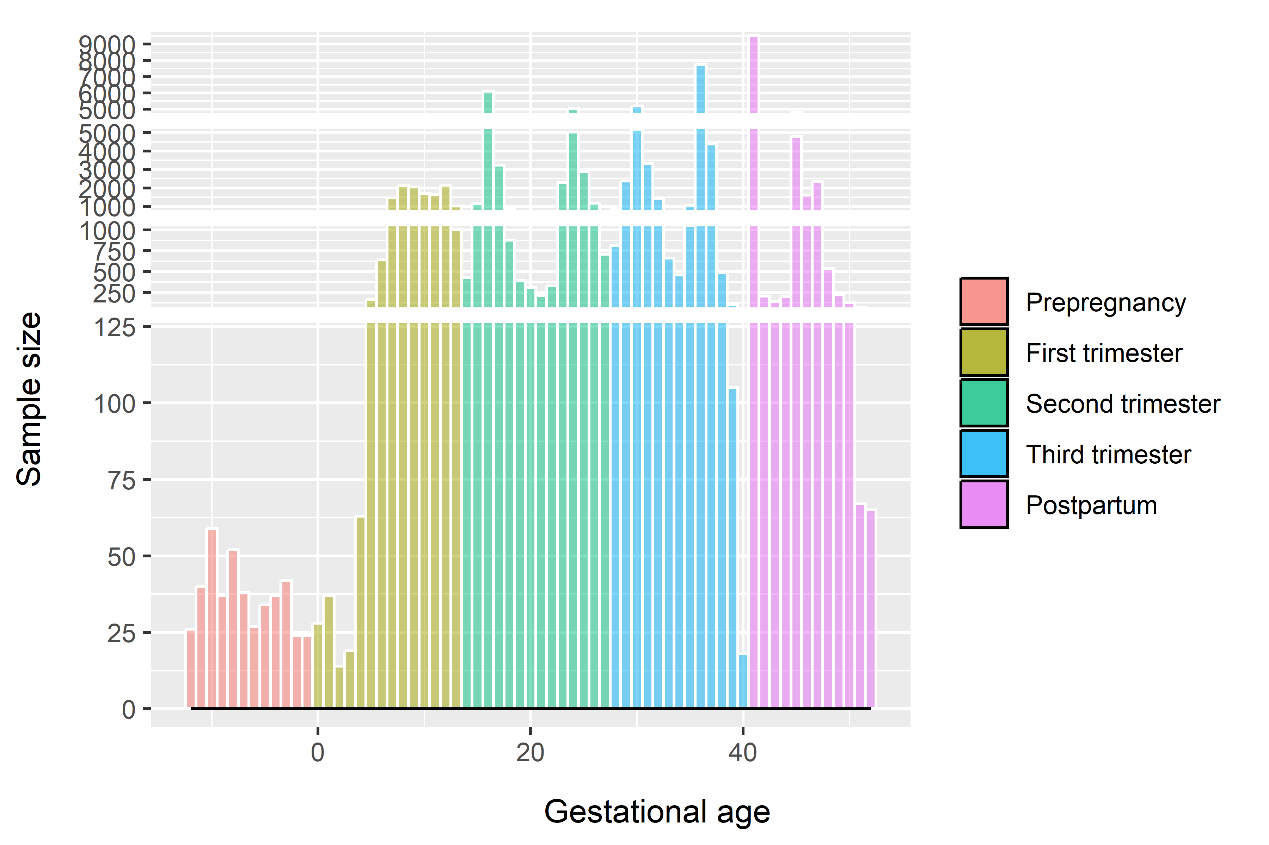


Figure S2. A combination of histogram and density curve for white blood cell count. White blood cell count is not normally distributed.


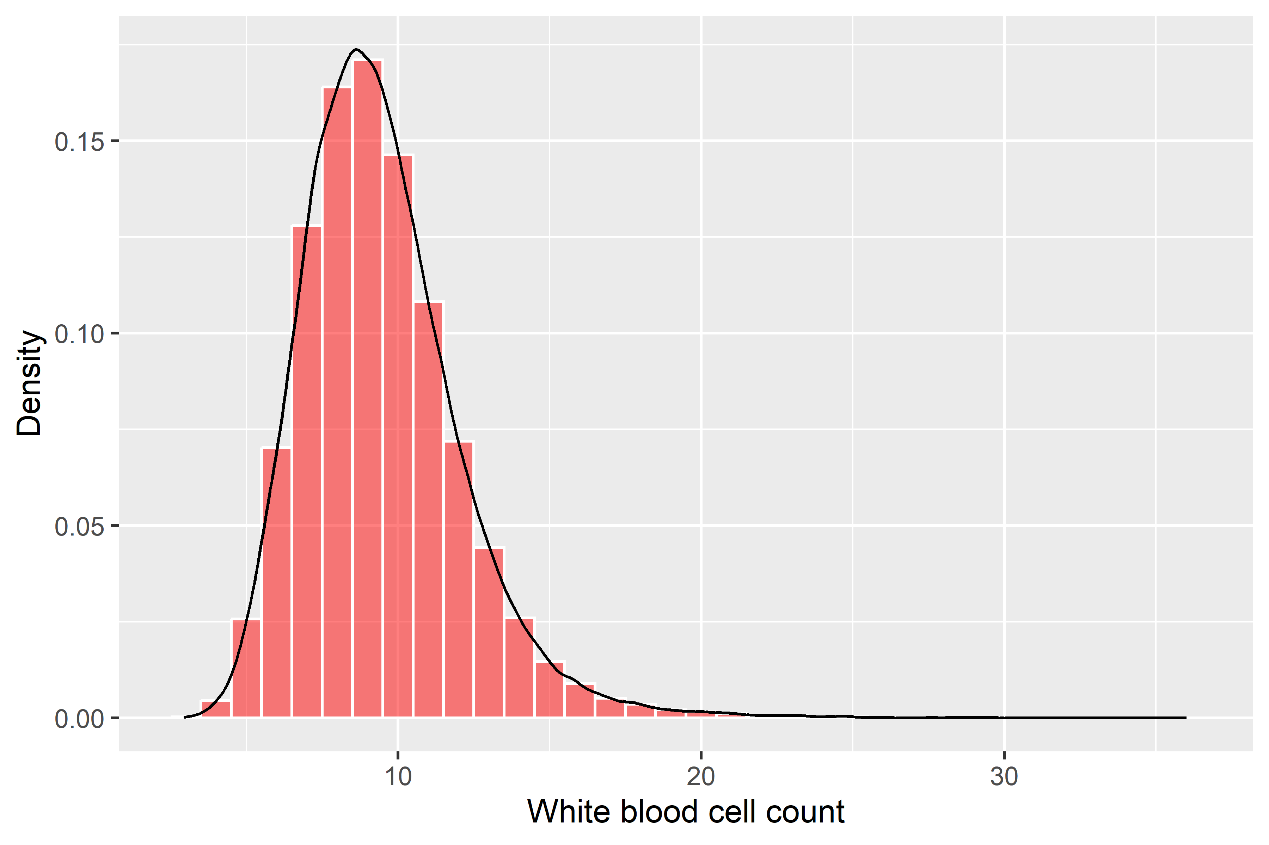


Figure S3. Log transformation of white blood cell. (a) Histogram and density curve show non-normal distribution of white blood cell. (b) Histogram and density curve show normal distribution of white blood cell after log transformation. (c) Trend of log-transformed white blood cell count across pregnancy. Medians and 95% confidence interval (light blue zone) in each week were displayed.


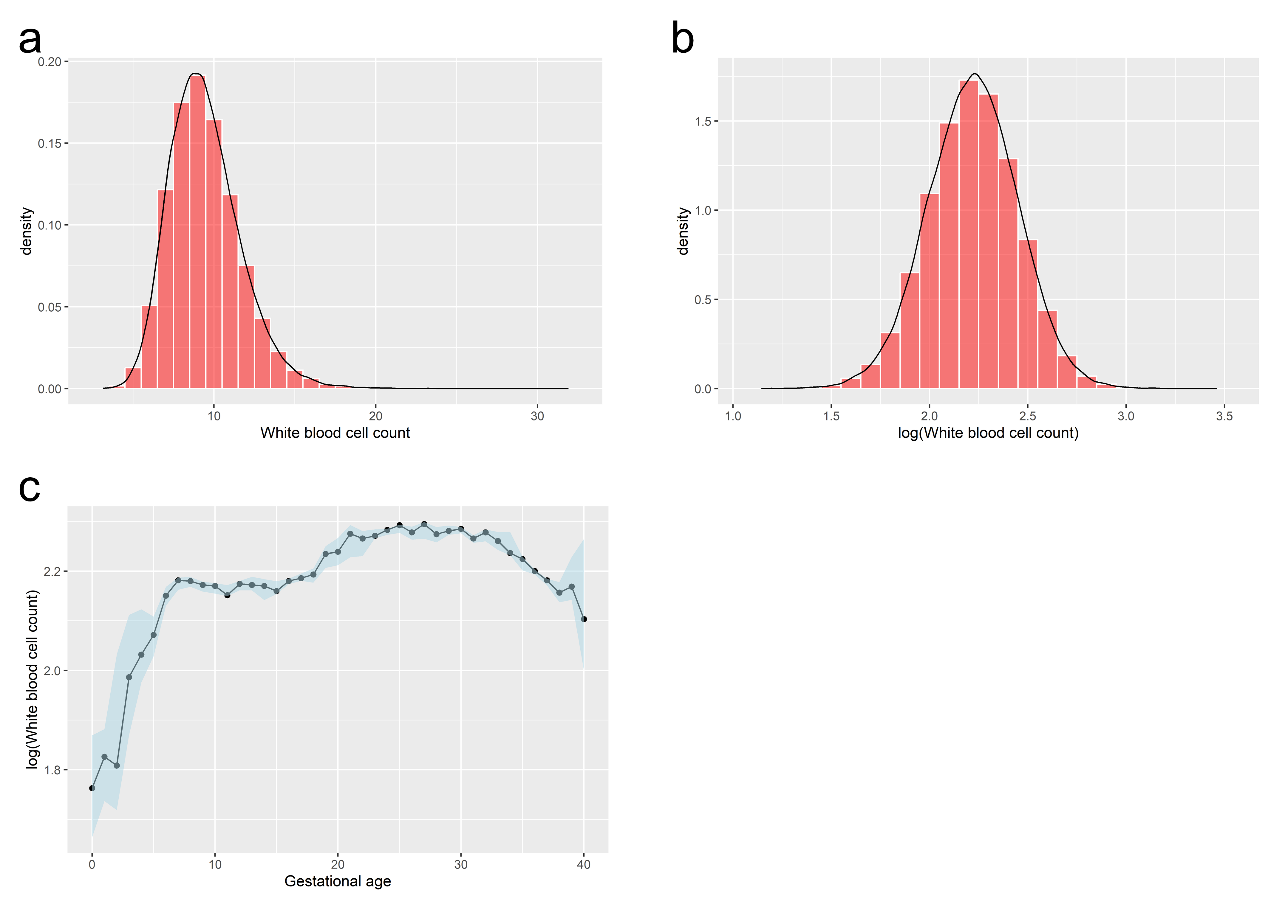


Figure S4. Trend of residuals from threshold regression model. The medians and 95% confidence interval (light blue zone) of absolute values of residuals in each gestational week were showed.


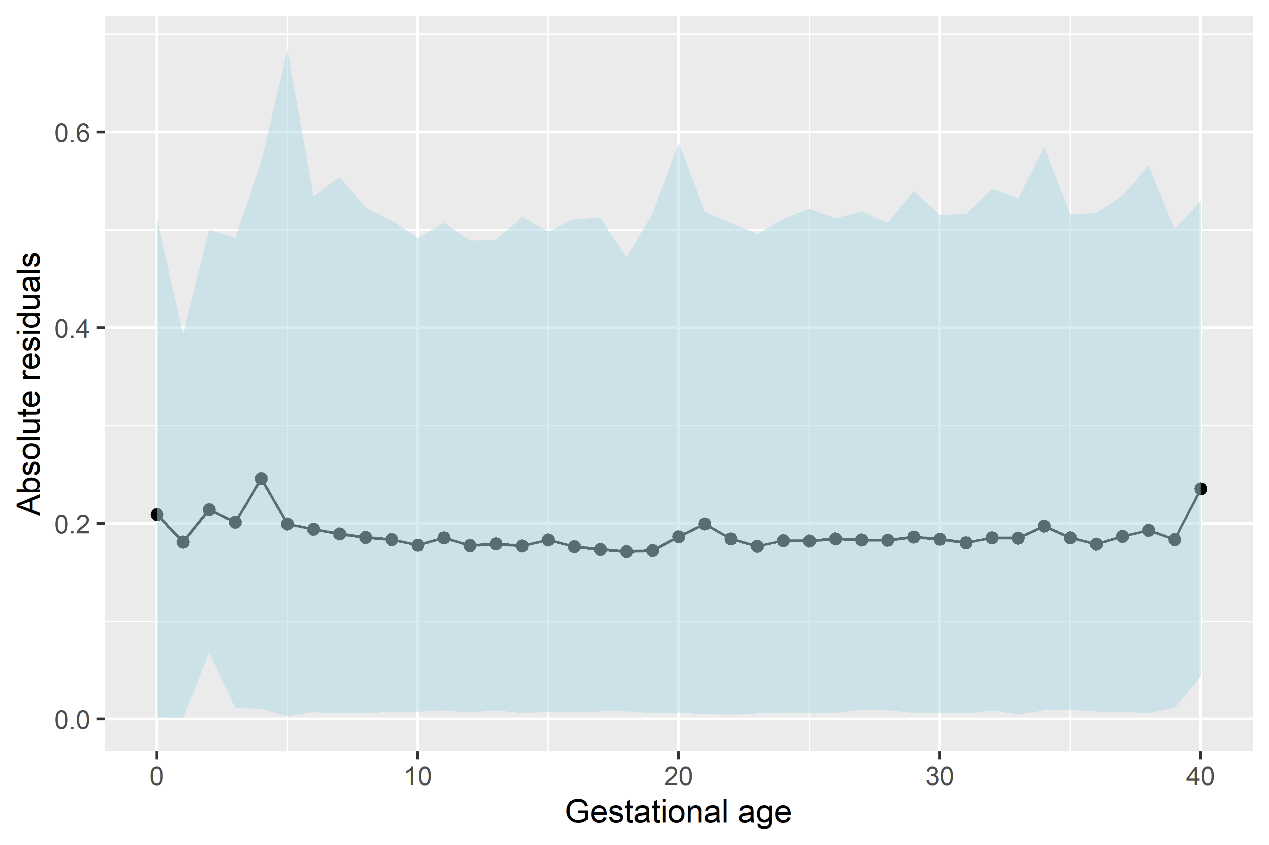


Figure S5. Comparisons of means and reference intervals for 7 to 40 weeks of gestation derived from two methods, threshold and linear regression. Two methods showed similar means and reference intervals for white blood cell count.


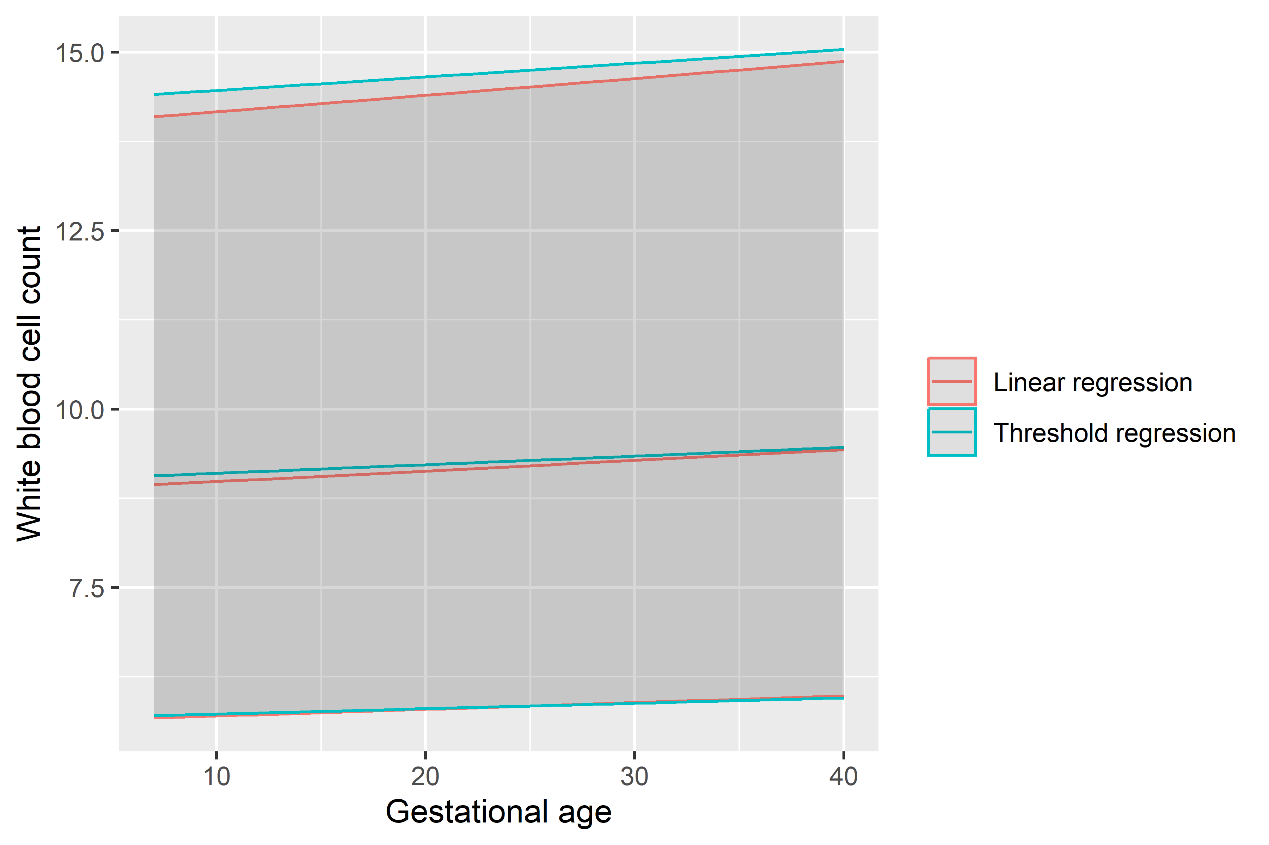


Figure S6. Comparison of the proportion of pregnant women with different count of complications between the High and non-High white blood cell count groups. The High group exhibited a higher proportion of women experiencing one, three, or four complications.


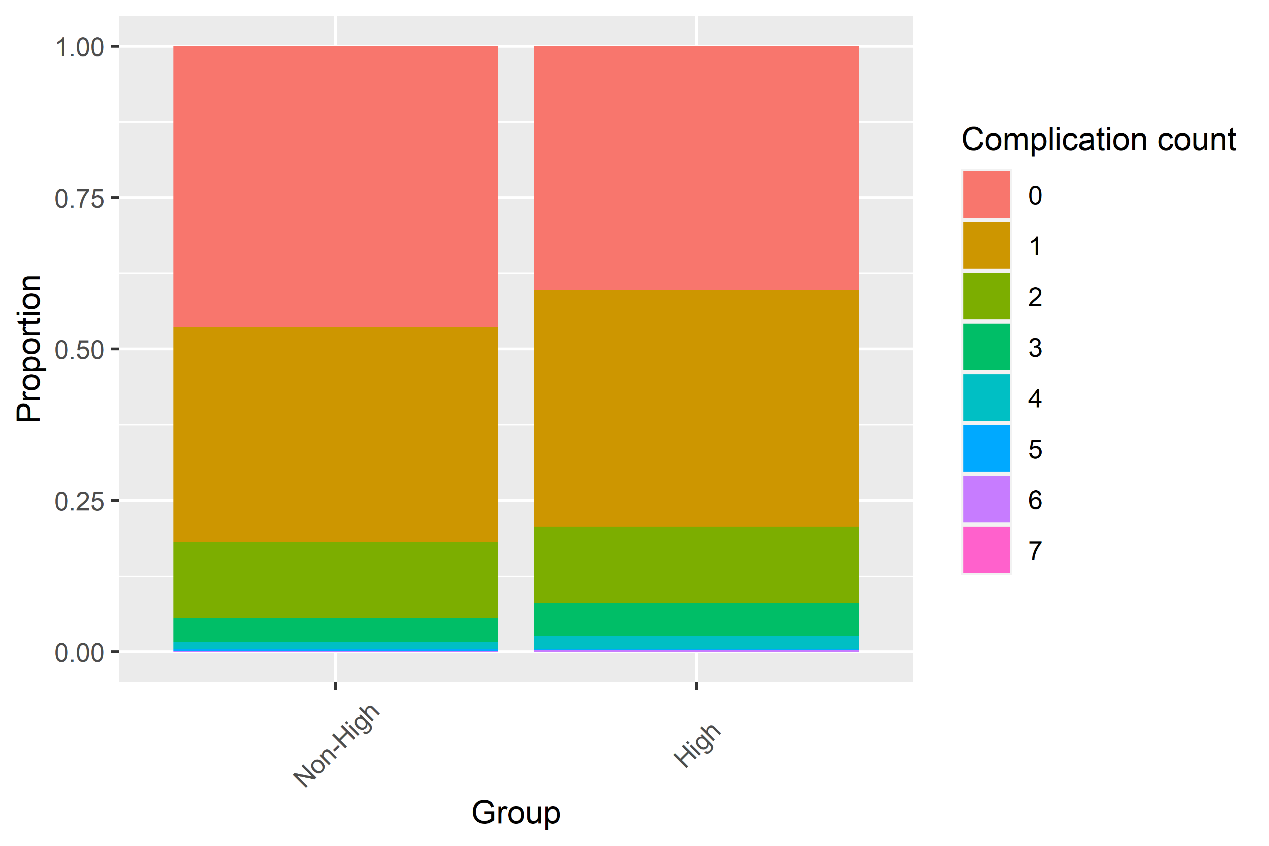


Table S1. Demographic and clinical characteristics of all subjects.

| Variable | All subjects  *n*= 17737 |
| --- | --- |
| Ethnicity: |  |
| Han, *n* (%) | 17221 (97.1%) |
| Minority, *n* (%) | 516 (2.91%) |
| Age (years), mean (SD) † | 30.2 (4.48) |
| BMI (kg/m2), mean (SD) | 21.0 (2.58) |
| Systolic blood pressure (mmHg), mean (SD) | 115 (7.44) |
| Diastolic blood pressure (mmHg), mean (SD) | 70.4 (6.01) |
| Gravidity, median (IQR) ‡ | 2.00 [1.00, 3.00] |
| Parity, median (IQR) | 1.00 [0.00, 1.00] |
| Gestational age (weeks), mean (SD) | 38.8 (1.51) |
| Delivery style: |  |
| Cesarean section, *n* (%) | 6182 (34.9%) |
| Natural birth, *n* (%) | 11555 (65.1%) |
| Neonatal sex: |  |
| Female, *n* (%) | 8400 (47.4%) |
| Male, *n* (%) | 9337 (52.6%) |

†SD: standard deviation. ‡IQR: interquartile range.

Table S2. Reference interval for each gestational week.

| Gestational week (week) | Reference intervals (×10^9/L) |
| --- | --- |
| 1 | 3.9-9.9 |
| 2 | 4.2-10.5 |
| 3 | 4.4-11.2 |
| 4 | 4.7-11.9 |
| 5 | 5-12.7 |
| 6 | 5.4-13.5 |
| 7 | 5.7-14.4 |
| 8 | 5.7-14.4 |
| 9 | 5.7-14.4 |
| 10 | 5.7-14.5 |
| 11 | 5.7-14.5 |
| 12 | 5.7-14.5 |
| 13 | 5.7-14.5 |
| 14 | 5.8-14.5 |
| 15 | 5.8-14.6 |
| 16 | 5.8-14.6 |
| 17 | 5.8-14.6 |
| 18 | 5.8-14.6 |
| 19 | 5.8-14.6 |
| 20 | 5.8-14.7 |
| 21 | 5.8-14.7 |
| 22 | 5.8-14.7 |
| 23 | 5.8-14.7 |
| 24 | 5.8-14.7 |
| 25 | 5.8-14.8 |
| 26 | 5.8-14.8 |
| 27 | 5.9-14.8 |
| 28 | 5.9-14.8 |
| 29 | 5.9-14.8 |
| 30 | 5.9-14.8 |
| 31 | 5.9-14.9 |
| 32 | 5.9-14.9 |
| 33 | 5.9-14.9 |
| 34 | 5.9-14.9 |
| 35 | 5.9-14.9 |
| 36 | 5.9-15.0 |
| 37 | 5.9-15.0 |
| 38 | 5.9-15.0 |
| 39 | 5.9-15.0 |
| 40 | 6.0-15.0 |
